# Construction and Functional Validation of a Cross-Niche Multifunctional Microbial Consortium for Straw-Returning Agricultural Systems

**DOI:** 10.3390/microorganisms14010135

**Published:** 2026-01-07

**Authors:** Shu Jia, Hang Qu, Bo Li, Jin Chu, Yinghua Juan, Yuehua Xing, Yan Liu, Hongjing Bao, Wentao Sun

**Affiliations:** 1 Institute of Plant Nutrition and Environmental Resources, Liaoning Academy of Agricultural Sciences, Shenyang 110161, China; jiashuin1987@126.com (S.J.); quhang8377@163.com (H.Q.); libocaas@163.com (B.L.); juanyong_001@sohu.com (Y.J.); lnzhsxyh@126.com (Y.X.); liuyan1980@163.com (Y.L.); baohjln@163.com (H.B.); 2 Institute of Plant Protection, Liaoning Academy of Agricultural Sciences, Shenyang 110161, China; anthor0601@163.com

**Keywords:** multifunctional microbial consortium, lignocellulose decomposition, enhanced nitrogen release, biocontrol, yield enhancement

## Abstract

Straw returning, a core practice in conservation tillage, promotes sustainable intensification; however, it faces challenges such as inefficient decomposition, nutrient competition, and pathogen accumulation. To address these limitations, this study aimed to develop a multifunctional microbial consortium specifically designed for straw-incorporating cropping systems. The consortium comprises four *Bacillus* strains with complementary enzymatic systems, isolated from diverse ecological niches. It exhibited robust lignocellulolytic enzyme production, with manganese peroxidase (7709.33 U/L), laccase (450.65 U/L), endo-β-1,4-glucanase (154.67 U/mL), and filter paper activity (309.18 U/L). The consortium significantly enhanced rice straw degradation by 37.18% and increased nitrogen (N) release by 16.13% compared to the control. Moreover, the consortium exhibited a 67.56% inhibition rate against *Magnaporthe oryzae* and reduced both the incidence rate and disease index of leaf blast and panicle blast. Field trials revealed increases in the rice grain yield of 9.63% and 6.94% when applied alone and 6.75% and 5.18% when co-applied with straw residues. These findings highlight the multifunctional agricultural potential of the consortium and provide a sustainable strategy to overcome the limitations of straw-incorporating farming systems.

## 1. Introduction

Crop straw is a highly valuable renewable resource, with global production reaching approximately 3.5 billion metric tons annually. It serves as an important source of organic fertilizer in agricultural ecosystems [1]. Straw return, a core measure in conservation tillage, mitigates the environmental pollution caused by open burning or improper disposal and reduces dependence on synthetic fertilizers, supporting sustainable agricultural intensification [2]. Previous studies have demonstrated that incorporating straw into soil enhances soil organic carbon sequestration, improves aggregate stability, and optimizes C–N cycling, thereby sustaining long-term soil fertility and productivity [3]. However, straw incorporation poses several technical challenges.

During straw decomposition, microbes compete with crops for nitrogen, leading to temporary nitrogen immobilization in the soil. Furthermore, the high carbon-to-nitrogen (C/N) ratio of straw reduces decomposition rates and nutrient release, resulting in a temporal mismatch between nutrient availability and crop demand [4]. This asynchrony causes transient nitrogen deficiency, which adversely affects crop growth and yield. Additionally, untreated crop straw may harbor pathogenic microorganisms and pest eggs. Straw return creates a favorable environment for certain plant pathogenic fungi by providing abundant nutrient substrates, promoting their proliferation [5]. For instance, a previous study reported that straw retention for three years significantly increased the abundance of *Magnaporthe oryzae* and *Ustilaginoidea virens* [6]. Similarly, long-term (10-year) crop residue retention significantly increased populations of *Fusarium graminearum* and *Fusarium moniliforme*, thereby increasing the risk of disease outbreaks [7].

The use of microbial inoculants can effectively alleviate these challenges. The combined application of crop straw and microbial decomposer inoculants enhances straw decomposition efficiency, facilitates nutrient release, and improves soil nutrient availability, thereby reducing microbial immobilization of soil nitrogen (N) [8]. Furthermore, the co-application of straw with biocontrol agents suppresses the relative abundance of phytopathogenic fungi and reduces seedling damage caused by pathogenic fungi [9]. Additionally, plant growth-promoting rhizobacteria (PGPR) enhance nutrient cycling through biological nitrogen fixation (e.g., *Rhizobium* spp. and *Azotobacter* spp.) and phosphate solubilization (e.g., *Pseudomonas* spp. and *Bacillus* spp.), thereby improving crop yields [10]. By systematically integrating these complementary functional traits, microbial consortia can be rationally engineered to effectively overcome the key limitations of straw-incorporating agricultural systems.

The development of multifunctional microbial consortia necessitates a prioritized examination of the functional compatibility among artificially combined strains. Furthermore, introduced strains are often subject to environmental stressors and competition from native microbiota, leading to target gene suppression and reduced functional performance. Based on the principles of multisource synergistic compensation (i.e., combining strains from different sources to enhance overall function) and enzymatic functional complementarity (i.e., using enzymes with non-overlapping activities for sequential substrate degradation), this study aimed to construct a novel multifunctional microbial consortium by integrating functional microorganisms from three distinct ecosystems (soil, insect gut, and animal biogas slurry). The performance of the consortium in lignocellulose decomposition, pathogen suppression, and yield enhancement was systematically evaluated. This study provides a sustainable strategy for the safe and efficient utilization of agricultural waste, contributing to the circular bioeconomy of agroecosystems.

## 2. Materials and Methods

### 2.1. Strains, Variety, and Experimental Site Description

*M. oryzae*, *Rhizoctonia solani*, *U. virens*, and *Fusarium fujikuroi* were provided by the Rice Disease Research Laboratory, Institute of Plant Protection, Liaoning Academy of Agricultural Sciences. The rice (*Oryza sativa* L.) cultivar utilized was Liaojing 327, and the rice seedlings were provided by the Liaoning Rice Research Institute, Liaoning Academy of Agricultural Sciences.

The study was conducted in Shimiaozhi Village (123°34′58″ E, 41°47′24″ N), Wangjia Township, Hunnan District, Shenyang City, Liaoning Province, China. This region has a temperate monsoon climate, with an average annual precipitation of 650 mm, a mean annual temperature of 6.2–9.7 °C, and a frost-free period of 155–180 d. The test soil was classified as hydragric anthrosol, with the following physicochemical properties in the surface layer (0–20 cm): pH, 5.81; soil organic carbon (SOC), 14.81 g/kg; total nitrogen (TN), 1.09 g/kg; total phosphorus (TP), 0.51 g/kg; total potassium (TK), 17.43 g/kg; alkali-hydrolyzable nitrogen (AN), 20.33 mg/kg; available phosphorus (AP), 7.40 mg/kg; and available potassium (AK), 71.10 mg/kg.

### 2.2. Screening of Straw Degradation Strains

Screening medium: Monoclonal strains from pure cultures were inoculated into Congo red cellulose medium and aniline blue medium [11] and cultured at 20 °C for 7 d. The colony diameter and the diameter of the surrounding transparent circle were measured, and the experiment was repeated three times.

The lignocellulolytic enzyme activity of a 1% inoculum of the candidate strain fermentation broth (10^7^ CFU/mL) was transferred into 100 mL of a lysogeny broth (LB) and potato dextrose agar (PDA) liquid medium. Cultures were incubated at 20 °C with shaking at 150 rpm for 3 d. A 5 mL sample of the solution was aseptically collected from each flask and centrifuged at 4 °C and 12,000× *g* for 20 min. The supernatant was collected for further analysis. The activities of manganese peroxidase (MnP, Kit catalog numbers: MNP-W96S-N), laccase (Kit catalog numbers: LAC-W48S-N), endo-β-1,4-glucanase (Kit catalog numbers: CL-W48S-N), and filter paperase (FPA, Kit catalog numbers: FPA-W48S-N) were measured according to the manufacturer’s instructions (Mlbio (Shanghai) Co., Ltd., Shanghai, China). As a comprehensive indicator of cellulose-degrading capability, FPA effectively reflects the strain’s overall cellulolytic potential. And MnP serves as the primary enzymatic system for lignin depolymerization, while laccase contributes to lignin decomposition, ultimately forming complex humic substances during straw decomposition processes [12]. Cellulase activity typically represents a synergistic multi-enzyme complex. In this study, we measured endo-β-1,4-glucanase activity, a key component of the native cellulase complex, using a commercial assay kit with carboxymethyl cellulose (CMC) as the substrate. One unit of enzyme activity (U) is defined as the amount of enzyme required to catalyze the production of 1 μg of glucose per minute per milliliter of culture filtrate. FPA reflects the synergistic action of multiple cellulase components, including endoglucanase, exoglucanase, and β-glucosidase. Enzymatic activity is defined as the amount of enzyme required to release 1 mg of glucose per minute from filter paper per milliliter of culture filtrate under the conditions of 50 °C and pH 4.6.

Straw decomposition rate: The weight-loss method was used to determine the degradation rate of rice straw by the candidate strains [13]. Rice straw was rinsed thoroughly with clean water and dried at 80 °C until a constant weight was achieved. A total of 10 g of dried straw was placed in a sterilized Erlenmeyer flask, followed by the addition of 10 mL of the candidate strain fermentation broth (10^7^ CFU/mL) and 100 mL of sterile water. The mixture was incubated at 20 °C for 15 d. The straw degradation rate was calculated as follows:

$$\mathrm{Straw}\;\mathrm{decomposition}\;\mathrm{rate}\;(\%)=\frac{Mo-Mt}{Mo}\times 100\%$$

where *Mo* is the dry weight of the added straw, and *Mt* is the dry weight of the straw at decomposition time *t*.

### 2.3. Screening of Antagonistic Strains Against Rice Pathogens

The plate confrontation method [14] was used to determine the antagonistic activity of the candidate strains against rice pathogens. Approximately 5 mm pathogen disks were placed at the center of a 90 mm diameter PDA plate, and bacteria were inoculated 25 mm from the center. In the controls, 5 mm pathogen disks were placed at the center of a 90 mm diameter PDA plate. Plates were cultured at 28 °C until the fungus in the control plate covered the entire plate. Antagonistic activity was evaluated using the inhibition rate (*IR*), calculated using the following formula:

$$\mathrm{IR}\;(\%)=\frac{\left(D-5)-(d-5\right)}{D-5}\times 100\%$$

where *D* is the diameter of the pathogen disks in the control treatment, *d* is the diameter of the pathogen disks with antagonistic bacteria, and 5 is the diameter (mm) of the disks inoculated with the pathogens.

### 2.4. Inter-Strain Antagonism Assessment

Candidate strains demonstrating strong antagonistic activity, high lignocellulolytic enzyme production, and high straw decomposition efficiency were selected for compatibility assessment. Bacterial interactions were analyzed using LB agar cross-streak assays, and fungal compatibility was assessed using a PDA dual culture. Bacterial–fungal interactions were examined through PDA co-cultivation, with inhibition zones as phenotypic indicators.

### 2.5. Construction of the Multifunctional Microbial Consortium

Seed cultures of the selected strains at 10^7^ CFU/mL were inoculated into LB liquid medium at a 1:1 ratio and incubated at 20 °C with shaking at 150 rpm for 3 d. Lignocellulolytic enzyme activities and straw degradation rates of the resulting microbial consortia were measured. The toxic medium method [15] was used to determine the antibacterial activity of the multifunctional consortium against pathogenic fungi.

### 2.6. Identification of Strains

Strains were identified based on morphological observations and molecular identification. Selected strains were inoculated onto LB plates using the continuous streaking method and incubated at 20 °C for 1 d. Colony growth and morphological characteristics were also recorded. The strains were identified using 16S rDNA and *gyrB* gene sequences. Genomic DNA was extracted using a bacterial genomic DNA FastPrep extraction kit (Sangon Biotech, Shanghai, China). DNA was quantified using a NanoDrop ND100 spectrophotometer (Thermo Fisher Scientific, Waltham, MA, USA) and stored at −20 °C. The primer sequences, polymerase chain reaction (PCR) mixtures, and conditions used are provided in Supplementary Table S1. The amplified products were sequenced by Sangon Biotech (Shanghai, China). The 16S rDNA and *gyrB* genes were sequenced using the Sanger method on an ABI 3730XL DNA analyzer (Foster City, CA, USA). The sequencing results were subjected to BLAST (https://www.ncbi.nlm.nih.gov/, accessed on 8 October 2025) analysis against nucleotide sequences from the GenBank database, using *Escherichia coli* as the external reference. Multiple sequence alignment with closely related *Bacillus* strains and similarity calculations were performed using CLUSTAL X [16]. Phylogenetic trees were constructed using MEGA 6.0 [17] using the neighbor-joining method with 1000 bootstrap replicates [18].

### 2.7. Pathogen Inhibition Test of the Microbial Consortium

Pot experiments were conducted in early April to assess the effectiveness of the microbial consortium in controlling *M. oryzae*, the causative agent of rice blast. The experimental treatments included (1) RS + *M. oryzae*: rice straw + *M. oryzae*, and (2) RS + *M. oryzae* + CG: rice straw + *M. oryzae* + microbial consortium. The pots used had dimensions of 40 cm (length) × 60 cm (width) × 28 cm (height). The rice straw application rate was set at 9000 kg·ha^−1^, which corresponded to 0.21 kg of straw per pot based on the pot area. The straw was finely chopped and inoculated with the pathogen at a concentration of 1 × 10^5^ CFU/mL, with an inoculation volume of 100 mL. Subsequently, the microbial consortium fermentation broth was inoculated at a concentration of 1 × 10^6^ CFU/mL, with an inoculation volume of 210 mL. The inoculated straw was thoroughly mixed with 40 kg of soil and placed into the pots.

Rice seedlings were transplanted on May 28, with two rows per pot, four hills per row, and three seedlings per hill. Soil samples from the three pots were collected at 10, 20, 30, 50, 70, 100, and 170 d post-inoculation for quantitative real-time PCR (qRT-PCR) analysis to determine the abundance of *M. oryzae*. The primer sequences, PCR systems, and conditions are listed in Supplementary Table S1. The incidence, disease index, and control efficacy of leaf blast and panicle blast were evaluated at the late tillering (70 d) and maturity (170 d) stages. The grading criteria for leaf blast and neck blast were adopted from Tan et al. [19]. The disease incidence rate (*DIR*), disease index (*DI*), and control efficacy (*CE*) were calculated as follows:

$$DIR=\frac{n}{N}\times 100;\;DI=\frac{\left[\Sigma (Ni\times i)\right]}{N\times 4}\times 100;\;CE\;(\%)=\frac{{DI}_{CK}-{DI}_{T}}{{DI}_{CK}}\times 100$$

where *n* is the number of infected plants, *N* is the total number of investigated plants, *Ni* is the number of infected plants at a given severity level, *i* is the severity level, *DI_CK_* is the disease index of the control, and *DI_T_* is the disease index of the treatment.

### 2.8. Functional Evaluation of the Microbial Consortium for Straw Degradation and Yield Enhancement

A field microplot experiment was conducted from April 22 to October 11. Four treatments were established: (1) CK, blank control; (2) RS, rice straw only; (3) CG, microbial consortium only; and (4) RS + CG, rice straw + microbial consortium. Each plot measured 5 × 6 m (30 m^2^), with three replicates per treatment. The rice straw application rate was set at 9000 kg·ha^−1^, with 27 kg of straw applied per plot. The rice straw was chopped into 3–5 cm segments and evenly spread on the surface of the paddy field. A composite microbial consortium was applied at a dosage of 1.25 L, followed by uniform application of 90 g of urea. Straw was incorporated into the soil through rotary tillage, and the field was subsequently flooded. Water was managed using alternating wet and dry irrigation practices. Transplantation was conducted on May 28 at a spacing of 30 cm × 18 cm. Next, 10 g of rice straw was placed in mesh bags and buried in the soil of treatment groups 2 and 4. At 10, 20, 30, 50, 70, 100, and 170 d, three treatments were randomly sampled to calculate the decomposition rates of rice straw [13] as well as the release rates of nitrogen (N), phosphorus (P), and potassium (K) [20,21]. During the tillering stage, plant height and tiller number were measured for ten fixed rice hills in each plot using a fixed-point sampling method. Harvesting was conducted on October 11. A 1 m^2^ area was randomly selected from each plot to determine rice yield. Additionally, three hills of rice plants were sampled to assess yield components, including plant height, effective panicle number, 1000-grain weight, grain number per panicle, and percentage of unfilled grains.

## 3. Results

### 3.1. Screening of Multifunctional Strains

**Figure 1:**
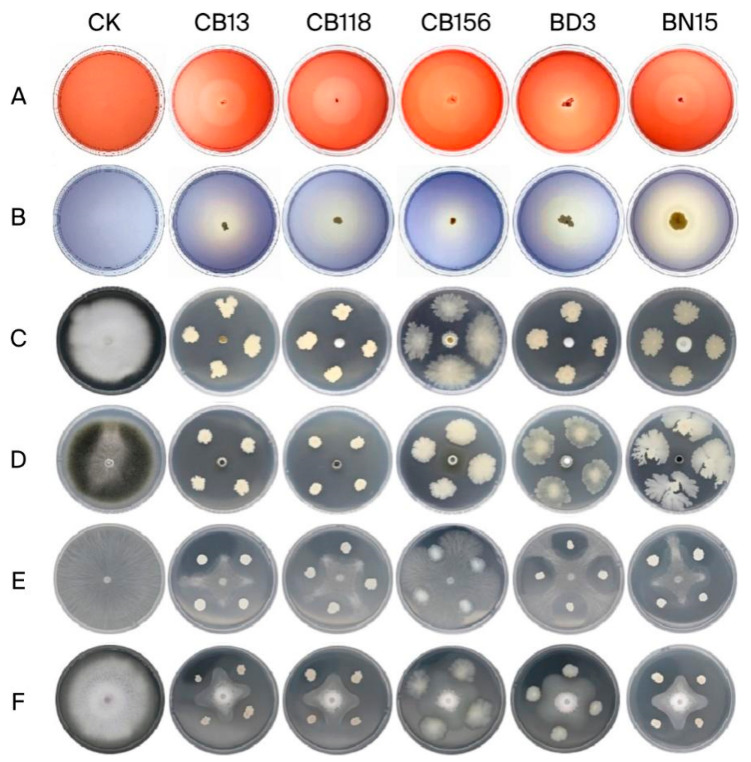
Colony and degradation cycles of some strains screened on cellulose Congo red medium (**A**) and aniline blue medium (**B**), and the antifungal effects of the partial strains against different rice pathogens. CK: blank control; (**C**) *Ustilaginoidea virens*; (**D**) *Magnaporthe oryzae*; (**E**) *Rhizoctonia solani*; (**F**) *Fusarium fujikuroi*.

Primary screening of 1295 bacterial and 32 fungal isolates on selective media identified 135 bacteria and 10 fungi that produced hydrolytic zones. Based on quantitative clearance zone measurements (Figure 1A,B; Supplementary Tables S2 and S3), strains exhibiting a decolorization halo diameter exceeding 40 mm on CMC-Na medium and 10 fungal strains were selected for secondary screening, and non-hydrolytic isolates were excluded from further characterization.

Secondary screening identified BD3 as the most potent cellulolytic strain (Table 1), showing peak filter paper activity (FPA: 306.80 U/L) and straw degradation efficiency (24.7%) in a 15 d continuous reaction, reflecting a 41.4% improvement over the control (17.47%). Strain BN15 exhibited exceptional ligninolytic specialization, showing maximal MnP (4405.33 U/L) and laccase (438.31 U/L) activities, whereas CB118 displayed superior endo-β-1,4-glucanase production (157.53 U/mL). The fungal isolates demonstrated comparatively reduced cellulolytic performance, which was consistent with their enzymatic activity profiles.

The bacterial strains CB13, CB118, CB156, BD3, and BN15 exhibited broad-spectrum inhibitory effects against major rice pathogens (Supplementary Table S4; Figure 1C–F). Strain CB13 exhibited the strongest antagonistic activity against rice pathogenic fungi, with suppression rates of 96.49% against *U. virens*, 92.68% against *M. oryzae*, 66.33% against *R. solani*, and 69.62% against *F. fujikuroi*. Notably, the fungal isolates showed no measurable antagonistic effects against the tested pathogens.

### 3.2. Inter-Strain Antagonism Assessment

Based on a comprehensive evaluation of cellulolytic activity, rice straw decomposition efficiency, and antifungal activity against major rice pathogens, BD3, BN15, CB156 and CB118 showed superior lignocellulolytic enzyme activity and straw degradation efficiency. Strains CB13 and PY1 exhibited strong antagonistic activity against rice pathogenic fungi. For fungal strains, FH2, FH5, FF2, and FF3 showed superior lignocellulolytic enzyme activity and straw degradation efficiency.

Therefore, six bacterial strains and four fungal strains were subjected to inter-strain antagonism assays (Supplementary Table S5). Widespread antagonistic interactions were observed between candidate bacterial and fungal strains. The results revealed that strain PY1 exhibited antagonistic activity against nine other candidate strains. Bidirectional antagonistic interactions between CB118 and CB13 were also observed. Based on these results, strains CB156, BD3, and BN15 were selected to establish a core microbial consortium that demonstrated complementary enzymatic profiles. Notably, despite their mutual antagonism, strains CB13 and CB118 were retained as candidate strains for optimized composite formulations because of their individual endo-β-1,4-glucanase and antifungal activities.

### 3.3. Construction of a Multifunctional Microbial Consortium

**Figure 2:**
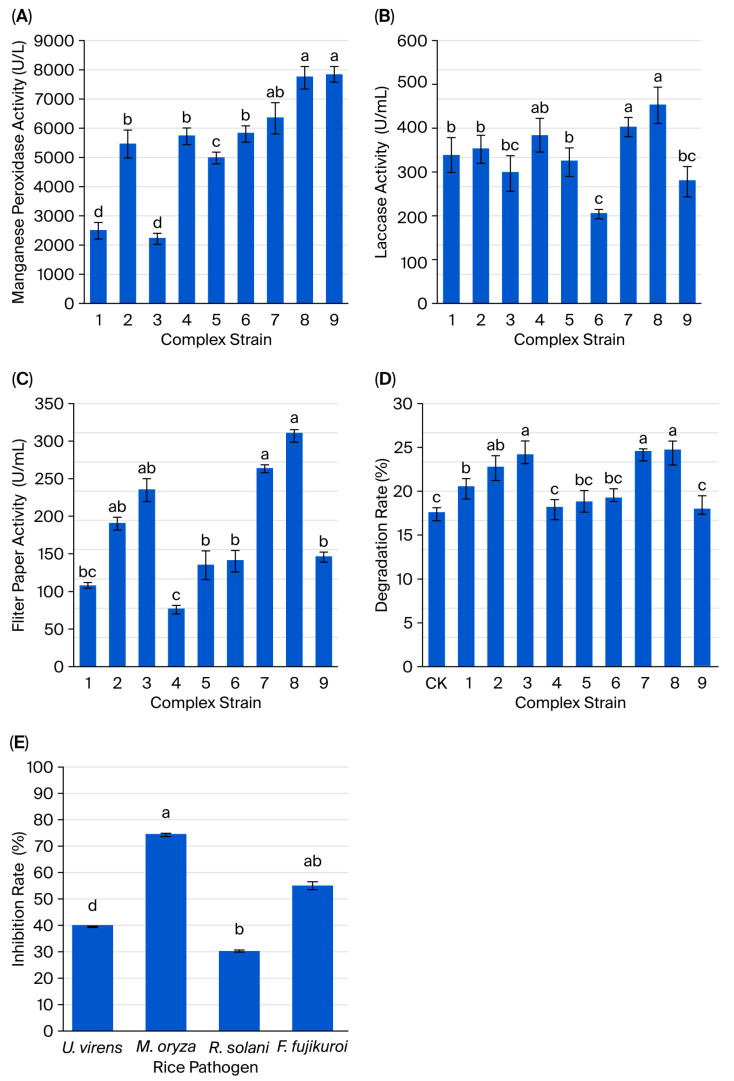
The enzymatic activities (**A**–**C**) and rice straw degradation rates (**D**) of different microbial consortia, and the inhibitory rates of the optimal microbial consortium against rice pathogenic fungi (**E**). (1) CB156; (2) BN15; (3) BD3; (4) BN15 + CB156; (5) BN15 + BD3; (6) CB156 + BD3; (7) BN15 + CB156 + BD3; (8) BN15 + CB156 + BD3 + CB13; and (9) BN15 + CB156 + BD3 + CB118. *U. virens*: *Ustilaginoidea virens*; *M. oryzae*: *Magnaporthe oryzae*; *R. solani*: *Rhizoctonia solani*; *F. fujikuroi*: *Fusarium fujikuroi*. Each value represents the mean ± standard error of values from three replicates per treatment. The different letters above the error bars indicate significant differences according to Duncan’s test (*p* < 0.05).

Strain consortium screening revealed that the CB156 + BD3 + BN15 consortium exhibited superior lignocellulolytic enzyme production, achieving 24.88% straw degradation (Figure 2). Subsequent supplementation with CB13 further enhanced the consortium’s enzymatic profile, yielding 7709.33 U/L (MnP), 450.65 U/L (laccase), 154.67 U/mL (endo-β-1,4-glucanase), and 309.18 U/L (FPA). This optimized consortium (CB156 + BD3 + BN15 + CB13) demonstrated a 24.90% straw degradation efficiency within 15 d, a 53.7% increase compared to the controls (16.20%). Notably, CB118 supplementation significantly impaired endo-β-1,4-glucanase activity (*p* < 0.05), FPA, and decomposition rates. Finally, CB156 + BD3 + BN15 + CB13 was identified as the optimal composition for the multifunctional microbial consortium. The sterile filtrates of the consortium showed broad-spectrum antifungal activity, suppressing pathogens by 73.95% (*M. oryzae*), 54.65% (*F. fujikuroi*), 39.56% (*U. virens*), and 29.98% (*R. solani*).

### 3.4. Identification of Strains

**Figure 3:**
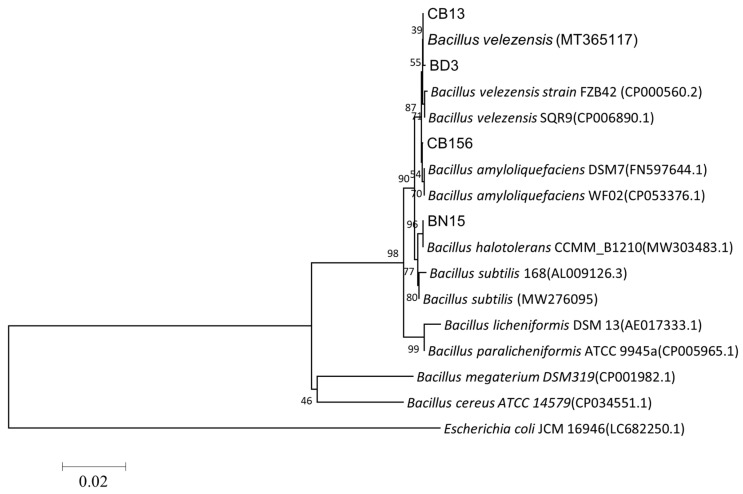
The phylogenetic tree based on 16S rRNA gene and *gyrB* gene sequence of the strains. The phylogenetic trees were constructed with the neighbor-joining method using the software package Mega, version 6.0, after multiple sequence alignments using Clustal X. GenBank accession numbers of bacterial strains are shown in parentheses. The number at each branch is the percentage of times the group of strains in that branch occurred, based on 1000 cycles, via bootstrap analysis.

Based on the culture characteristics, colony morphology on LB medium (Table 2), and neighbor-joining phylogenetic tree based on 16S rRNA gene and *gyrB* (Figure 3). The phylogenetic analysis based on the NCBI BLAST alignment revealed that strain BD3 clustered with *B. velezensis*, strain CB156 with *Bacillus amyloliquefaciens*, and strain BN15 with *Bacillus halotolerans*. Accordingly, the strains were taxonomically designated *B. velezensis* BD3, *B. amyloliquefaciens* CB156, and *B. halotolerans* BN15. Strain CB13 has been previously identified as *Bacillus velezensis* based on prior characterization [22]. The gene sequences of strains CB156, BD3, and BN15 were deposited in GenBank (accession numbers listed in Table 2).

### 3.5. Pathogen Inhibition by the Microbial Consortium Against M. oryzae

**Figure 4:**
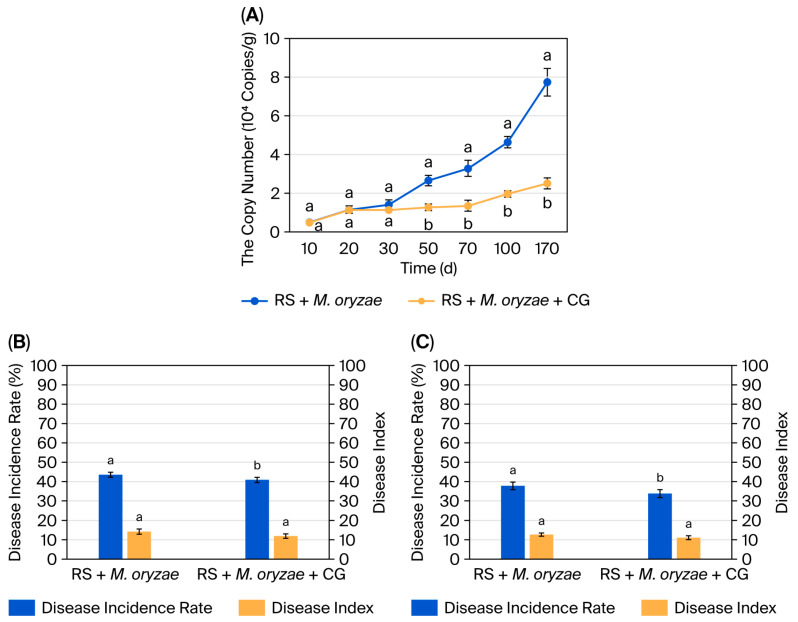
The inhibition rate of the microbial consortium against *Magnaporthe oryzae* (**A**) and the control efficacy against leaf blast (**B**) and panicle blast (**C**). Each value represents the mean ± standard error of values from three replicates per treatment. The different letters above the error bars indicate significant differences according to Duncan’s test (*p* < 0.05). RS+ *M. oryzae*: rice straw + *M. oryzae*; RS+ *M. oryzae* + CG: rice straw + *M. oryzae* + microbial consortium.

During the initial 10–30 d of straw incorporation, no significant difference in *M. oryzae* genomic copy number was observed between the two treatments (*p* > 0.05; Figure 4A). However, from 50 to 170 d of straw incorporation, the RS + *M. oryzae* + CG treatment resulted in a significantly lower *M. oryzae* genomic copy number than the RS + *M. oryzae* treatment (*p* < 0.05), demonstrating strong suppression of *M. oryzae* in the straw by the microbial consortium. By day 170, the inhibition rate had reached 67.57%.

The microbial consortium treatment also reduced the incidence rate and disease index of leaf blast and panicle blast (Figure 4B,C). At the late tillering stage, the incidence rate and disease index of leaf blast were 43.23% and 14.28% in the RS + *M. oryzae* treatment, whereas the RS + *M. oryzae* + CG treatment showed lower values (40.63% and 11.66). At the maturity stage, the incidence rate and disease index of panicle blast were 37.58% and 16.63% in the RS + *M. oryzae* treatment, compared with 33.54% and 10.88% in the RS + *M. oryzae* + CG treatment.

### 3.6. Straw Decomposition by the Microbial Consortium

**Figure 5:**
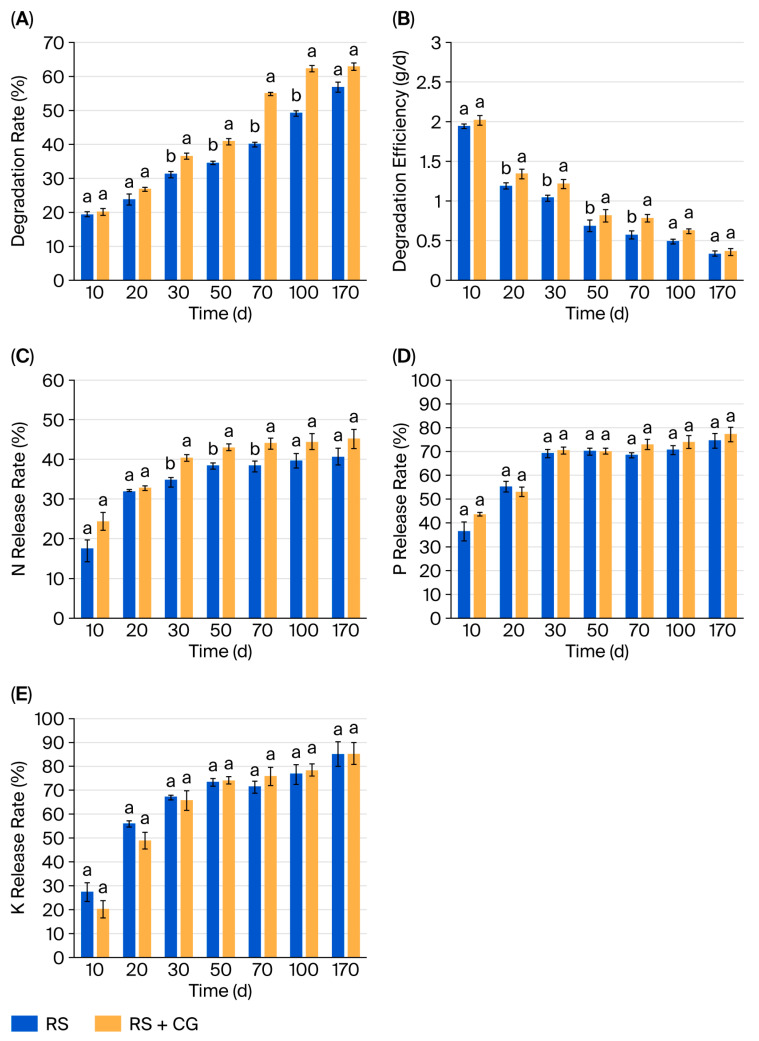
Degradation rates (**A**) and efficiency (**B**) of straw components by the composite microbial consortium, and the release rate of N (**C**), P (**D**), and K (**E**) from straw degradation. Each value represents the mean ± standard error of values from three replicates per treatment. The different letters above the error bars indicate significant differences according to Duncan’s test (*p* < 0.05). RS: rice straw only; RS + CG: rice straw + microbial consortium.

During the tillering and heading stages (30–100 d), straw degradation in the RS + CG treatment was significantly higher than that in the control (RS) (*p* < 0.05; Figure 5A,B). At peak decomposition (day 70), the RS + CG treatment achieved a 54.87% degradation rate, an increase of 14.87 percentage points over RS (a 37.18% improvement over the control). By maturity (day 170), decomposition rates reached statistically equivalent levels (RS + CG: 62.77% vs. RS: 59.80%, *p* > 0.05). During the initial decomposition phase (10 days post incorporation), no significant treatment-related differences in degradation efficiency were observed (*p* > 0.05). However, the RS + CG treatment exhibited significantly enhanced degradation efficiency compared with RS during the active decomposition phase (20–70 d) (*p* < 0.05). This period corresponds to accelerated microbial activity and cellulose breakdown. In the later stabilization phase (100–170 d), degradation rates progressively declined, with no significant inter-treatment differences observed (*p* > 0.05), suggesting the convergence of decomposition processes as labile fractions were depleted.

Straw nutrient analysis demonstrated that the RS + CG treatment significantly increased the nitrogen (N) release rate compared to the RS treatment (*p* < 0.05; Figure 5C–E), whereas no statistically significant effects were observed on the release rates of phosphorus (P) or potassium (K) (*p* > 0.05). The microbial consortium accelerated N release, achieving a cumulative release rate of 40.42% by day 30, accounting for 89.38% of the total N release. During days 30–70, the N release rate in the RS + CG treatment was significantly higher than in the control, exceeding it by 4.66–5.75 percentage points (a 12.16–16.13% increase). This early-phase nutrient availability facilitates N supply during critical growth stages (tillering and heading) in rice. By day 170, the final N release rate had reached 45.22% in the RS + CG treatment, compared to 40.51% in the control.

### 3.7. Crop Yield Promotion by the Microbial Consortium

**Figure 6:**
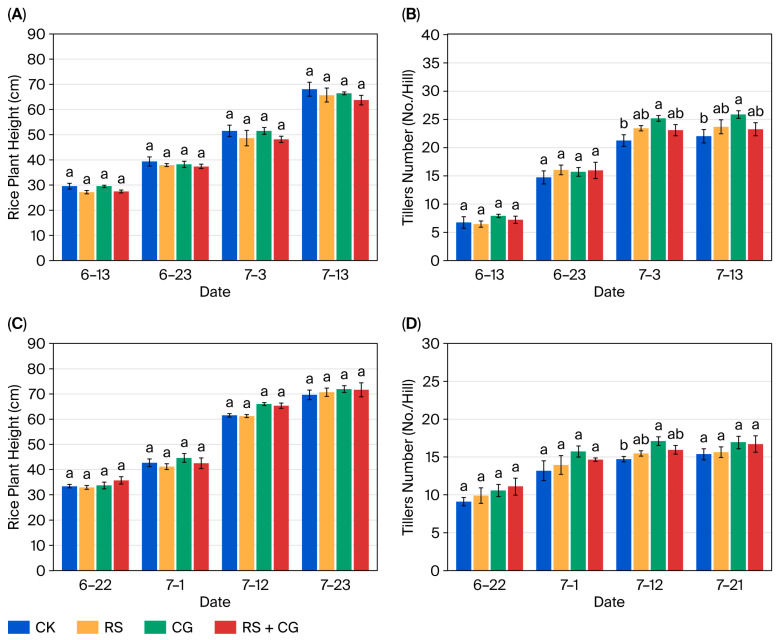
Dynamic changes in rice plant height (**A**,**C**) and tiller numbers (**B**,**D**) under different treatments. Each value represents the mean ± standard error of values from three replicates per treatment. The different letters above the error bars indicate significant differences according to Duncan’s test (*p* < 0.05). CK: blank control; RS: rice straw only; CG: microbial consortium only; RS + CG: rice straw + microbial consortium.

Rice growth parameters were monitored during the tillering stage (Figure 6). Although no significant differences in plant height were observed across the treatments (*p* > 0.05), the CG treatment significantly increased tiller production compared to the control (*p* < 0.05). In 2024, at the late tillering stage, the CG plants developed 25.9 tillers per hill versus 22.0 in the CK plants, representing a 17.7% increase. Although both RS and RS + CG treatments produced numerically more tillers than CK, these differences were not statistically significant (*p* > 0.05). In 2025, no significant differences in tiller number were detected across treatments (*p* > 0.05).

The yield performance analysis (Table 3 and Table 4) revealed that the CG treatment achieved the highest agronomic productivity, demonstrating 9.63% and 6.94% higher grain yields and effective panicle numbers, respectively, than CK. These results confirm the growth-promoting and yield-enhancing potential of the microbial consortium. By contrast, straw incorporation without microbial amendment (RS) resulted in the lowest productivity metrics. This reduction was attributed to the temporal overlap between straw decomposition and critical rice growth stages (tillering and heading), which induced microbial–crop competition for nitrogen, leading to nitrogen limitation during panicle initiation. However, later-stage nutrient release from straw decomposition improved grain-filling parameters (grains per panicle and 1000-grain weight). These compensatory effects partially offset the early growth penalty, resulting in a 2.08% and 5.68% yield reduction relative to CK. Importantly, microbial inoculation (RS + CG) effectively mitigated these adverse effects, increasing effective panicle numbers and providing a 6.75% and 5.18% yield improvement over the straw-only treatment through optimized nutrient cycling.

## 4. Discussion

### 4.1. Construction of a Multifunctional Microbial Consortium

Screening multifunctional microbial strains is a pivotal step in the formulation of multifunctional composite microbial consortia. Three *Bacillus* spp. strains with high-efficiency cellulose/lignin degradation and broad-spectrum antagonism against phytopathogenic fungi were isolated from long-term straw-return soils using selective enrichment and dual-culture confrontation assays [23]. The exogenous degradative bacterium ZJW-6 enhanced cellulose degradation and promoted positive bacterial–fungal interactions and enriched key microbial taxa associated with straw degradation, ultimately improving the degradation rate and promoting rice growth and development [24]. In the present study, we used a functional screening strategy targeting lignocellulose decomposition and pathogen inhibition to obtain highly efficient multifunctional microbial strains.

Strains CB156, BD3, BN15, and CB13 were isolated from three distinct ecological niches: long-term straw-amended agricultural soil, pig biogas slurry, and the intestinal tracts of *Antheraea pernyi*. These environments, characterized by unique physicochemical properties, facilitate microbial enrichment and functional specialization, making them highly enriched reservoirs of diverse functional microbiota. In soil ecosystems, straw return promotes significant enrichment of cellulose-degrading microorganisms, establishing soil as a natural reservoir for lignocellulolytic bacteria, such as *Trichoderma* and *Bacillus* species, which have been widely studied for their ability to effectively degrade straw [25]. Strains BD3 and CB156 were isolated from paddy field soil with a multi-year history of straw incorporation and demonstrated superior FPA activity. Biogas slurry, a primary byproduct of the anaerobic digestion of livestock manure, is widely used as a bio-organic fertilizer because it is rich in essential nutrients, including minerals, vitamins, hormones, and amino acids [26]. Additionally, it contains diverse antimicrobial compounds that exhibit inhibitory effects against over 50 phytopathogenic species and demonstrate field efficacy in terms of controlling various plant diseases [27]. The CB13 strain, isolated from swine biogas slurry, has been confirmed to exhibit significant antagonistic activity against multiple phytopathogens [22]. Numerous plant-feeding insects (e.g., grubs, mealworms, termites, and grasshoppers) are enriched with specialized lignocellulolytic bacterial communities in their gut [28,29,30,31]. *Bacillus* species isolated from white grub (*Holotrichia parallela*) and grasshopper guts have demonstrated significant potential for agricultural residue degradation [32,33]. Similarly, termite gut isolates, including *Bacillus* sp. BMP01 [34], and the fungal strains *Aspergillus nomius* (MN700028) and *Trichoderma harzianum* (MN700029), exhibit superior lignocellulose degradation capabilities [35]. The BN15 strain isolated from the gut of *A. pernyi* exhibited significant MnP and laccase activities, indicating its potential as a key microbial candidate for lignin biodegradation.

Bacteria, fungi, and actinomycetes all possess the ability to synthesize lignocellulolytic enzymes [25,36]. However, bacterial strains predominate in straw degradation systems because of their shorter generation times, faster enzyme production kinetics during fermentation, and the superior properties of bacterial cellulases, including broader substrate versatility and enhanced operational stability [37]. In the present study, all strains used to construct the microbial consortium belonged to *Bacillus* spp.

*Bacillus* species exhibit exceptional environmental adaptability, enabling their survival across diverse ecological niches [38]. Furthermore, genomic analyses indicate that approximately 5% of *Bacillus* genomes are dedicated to biosynthetic gene clusters (BGCs) responsible for bioactive compound production [39]. *Bacillus*-derived antimicrobial peptides show potent antimicrobial activity against various pathogenic bacteria and fungi [40]. *Bacillus velezensis* CH1 exhibits plant growth-promoting traits, including indole-3-acetic acid (IAA) production, biofilm formation, nitrogen fixation, and siderophore synthesis [41]. These findings highlight the significant potential of *Bacillus* spp. as multi-role agents for both biological control and biofertilization.

The synthetic microbial consortium constructed by combining bacterial strains from distinct ecological niches exhibited significantly enhanced metabolic activity and functional performance compared to the single-strain consortium. Similarly, a microbial consortium constructed from strains isolated from animal feces, soil, and straw samples achieved a rice straw degradation rate of 73.2% within 8 d, demonstrating significantly higher efficiency compared to individual strains [42].

### 4.2. Functional Validation of a Multifunctional Microbial Consortium

Conventional microbial consortia typically have functional limitations. Biocontrol agents demonstrate strong antimicrobial activity but poor straw degradation, whereas specialized lignocellulose-degrading consortia show only modest antimicrobial activity and suboptimal field performance [13]. Notably, the direct combined application of different functional consortia is frequently hindered by inter-strain antagonism. The development of multifunctional microbes offers effective solutions to these problems. Zhao et al. [37] created HY-1, a composite microbial consortium that exhibited synergistic nitrogen fixation, lignocellulose degradation, and plant growth promotion. Based on the straw-degrading and phosphorus-solubilizing capabilities of its strains, the constructed microbial consortium achieved a straw degradation rate of 48.3% within 7 d (a 7% increase compared to monocultures) and solubilized 117.54 mg·L^−1^ of insoluble phosphorus (a 29.81% increase in phosphorus-solubilizing efficiency) [8]. The microbial consortium developed in this study demonstrated three key benefits: (1) it enhanced straw decomposition by 14.87% (37.18% increase over control); (2) it exhibited a 67.56% inhibition rate against *M. oryzae*; and (3) it led to significant improvements in rice grain yield (9.63% and 6.94% yield enhancement when applied alone, and 6.75% and 5.18% increases when co-applied with straw residues). These findings demonstrate the multifunctional potential of this consortium for sustainable agriculture.

## 5. Conclusions

We developed a novel multifunctional microbial consortium for a straw-incorporating agricultural system through the strategic integration of complementary microorganisms from three ecological niches, effectively overcoming inefficient straw decomposition, pathogen accumulation, and yield instability. This study provides a sustainable strategy for safe and efficient utilization of agricultural waste. However, the mechanisms underlying multifunctional coupling and the ecological strategies that maintain functional stability under environmental stress remain to be elucidated.

## Figures and Tables

**Table 1:** Enzyme activities and straw degradation rates of partial strains.

Isolates	MnP (U/L)	Laccase (U/L)	endo-β-1,4-glucanase (U/mL)	FPA (U/L)	Straw Degradation Rate (%)
CB118	275.33 ± 0.00	222.24 ± 21.38	157.53 ± 11.96	120.88 ± 16.57	21.50 ± 0.44
BD3	1101.33 ± 275.22	246.93 ± 37.55	81.28 ± 2.81	306.80 ± 41.09	24.70 ± 0.35
BN15	4405.33 ± 430.08	438.31 ± 12.35	81.38 ± 4.14	181.47 ± 21.85	22.90 ± 0.38
CB156	1376.67 ± 275.22	327.19 ± 34.37	69.02 ± 12.78	236.71 ± 30.62	23.60 ± 0.67
CB13	0.00	141.99 ± 6.17	139.81 ± 2.12	116.13 ± 26.48	19.00 ± 0.85

MnP: manganese peroxidase; FPA: filter paperase.

**Table 2:** Colony morphology and identification results.

Strain Number	Colony Characteristics	Colony Morphology	The NCBI Registration Number	Strain Identified
CB13	Oval Milky-white Opaque Wrinkled surface Smooth edges	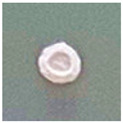	16S rRNA: OP430814 *gyr B*: OP889277	*Bacillus velezensis*
CB156	Oval Milky-white Opaque Smooth edges	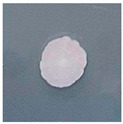	16S rRNA: PP809059 *gyr B*: PX767074	*Bacillus amyloliquefaciens*
BD3	Oval White Opaque Wrinkled surface Smooth edges	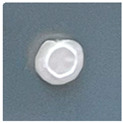	16S rRNA: PP033752 *gyr B*: PX767075	*Bacillus velezensis*
BN15	White Opaque Wrinkled surface Irregular edges	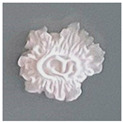	16S rRNA: PP809060 *gyr B*: PX767076	*Bacillus halotolerans*

**Table 3:** Effects of different treatments on rice yields and yield components (2024).

Treatment	Effective Panicles (×10^4^/hm^2^)	Grains per Panicle (No.)	1000-Grain Weight (g)	Seed Setting Rate (%)	Yield (kg/hm^2^)	Percentage Yield Increase (%)
CK	442.77 ± 4.82 a	105.11 ± 10.72 a	21.65 ± 0.27 a	93.44 ± 0.64 a	9201.18 ± 280.23 b	-
RS	402.39 ± 10.87 b	115.02 ± 6.38 a	22.26 ± 0.70 a	92.82 ± 0.98 a	9009.93 ± 272.30 b	−2.08
CG	448.95 ± 11.53 a	109.42 ± 4.62 a	21.61 ± 0.58 a	93.38 ± 0.69 a	10,086.87 ± 113.33 a	9.63
RS + CG	429.20 ± 13.42 ab	118.88 ± 7.27 a	21.77 ± 0.18 a	92.44 ± 1.21 a	9822.52 ± 116.13 a	6.75

CK: blank control; RS: rice straw only; CG: microbial consortium only; and RS + CG: rice straw + microbial consortium. Means followed by a different letter in the columns indicate significant differences according to one-way analysis of variance (*p* < 0.05).

**Table 4:** Effects of different treatments on rice yields and yield components (2025).

Treatment	Effective Panicles (×10^4^/hm^2^)	Grains per Panicle (No.)	1000-Grain Weight (g)	Seed Setting Rate (%)	Yield (kg/hm^2^ )	Percentage Yield Increase (%)
CK	413.63 ± 13.34 ab	108.24 ± 9.34 a	22.03 ± 0.89 a	94.37 ± 1.39 a	9028.82 ± 188.89 a	-
RS	386.72 ± 8.03 b	116.93 ± 10.43 a	22.67 ± 0.65 a	93.78 ± 1.89 a	8462.15 ± 229.33 b	−5.68
CG	420.03 ± 11.03 a	109.89 ± 7.78 a	21.98 ± 0.98 a	94.56 ± 1.47 a	9721.40 ± 176.30 a	6.94
RS + CG	397.78 ± 9.37 b	120.13 ± 8.37 a	22.86 ± 0.29 a	93.58 ± 0.96 a	9545.11 ± 142.53 a	5.18

CK: blank control; RS: rice straw only; CG: microbial consortium only; and RS + CG: rice straw + microbial consortium. Means followed by a different letter in the columns indicate significant differences according to one-way analysis of variance (*p* < 0.05).

## Data Availability

The original contributions presented in this study are included in the article/Supplementary Materials. Further inquiries can be directed to the corresponding author.

## Supplementary Materials

The following supporting information can be downloaded at: https://www.mdpi.com/article/10.3390/microorganisms14010135/s1, Table S1: Sequences of primers and PCR conditions used in this study; Table S2: The strains selected for secondary screening formed decolorization circles on Congo red-cellulose; Table S3: The strains selected for secondary screening formed decolorization circles on aniline blue media; Table S4: Inhibition rates of partial strains against rice pathogens. Table S5: Antagonistic test among candidate strains.
