# Supplementary material for: Construction and Functional Validation of a Cross-Niche Multifunctional Microbial Consortium for Straw-Returning Agricultural Systems
Source: Microorganisms. 2026 Jan 7;14(1):135. doi: 10.3390/microorganisms14010135 (PMC12843952; doi:10.3390/microorganisms14010135)
Supplement: Supplementary file 1 [file microorganisms-14-00135-s001.zip › microorganisms-4035467-supplementary.pdf]

Supplementary Table S1. Sequences of primers and PCR conditions used in this study

Target	Primer name	Sequence(5′-3′)	PCR conditions	PCR system
Magnaporthe oryzae	28SMF	ACCCTACTGATGACCTCG	95°C, 5min, 35 cycles × (95 °C, 30 s; 58 °C, 30 s; 72 °C, 1 min), kept at 4°C	The 20 μL polymerase chain reaction (PCR)-system: 10 μLof 2X Taq Plus Master Mix (2X), 0.8 μL of each primers (5 μM), 1 μL genomic DNA template (20 ng/μL DNA), and 7.4 μL deionized distilled water.
	28SMR	GTGTCAAAATTACAATACGC		
16S rDNA	27F	AGAGTTTGATCMTGGCTCAG	94°C, 5min, 30 cycles (95°C, 1min; 57°C, 30s; 72°C, 1min) , 72°C, 5min, kept at 4°C	The 50μL polymerase chain reaction (PCR)-system: 5μL of 10× PCR buffer, 2μL dNTPs, 1μL Pfu DNA polymerase (5 U/ μL), 3μL genomic DNA template (25ng/μL DNA), 2μL of each primers (0.1μM), and 35μL deionized distilled water.
	1492R	GGTTACCTTGTTACGACTT		
Gyr B	F	-TTATCTACGACCTTAGACG	94 °C, 5 min, 30 cycles (95 °C, 1 min; 58 °C, 30 s; 72 °C, 1 min), 72 °C, 5 min, kept at 4 °C.	The 50 μL polymerase chain reaction (PCR)-system contained 5 μL of 10× PCR buffer, 2 μL dNTPs, 1 μL Pfu DNA polymerase (Takara Bio Inc., Beijing, China), 3 μL genomic DNA template (25 ng/μL DNA), 2 μL of each primers (0.1 μM), and 35 μL deionized distilled water.
	R	TAAATTGAAGTCTTCTCCG		

Supplementary Table S2. The strains selected for secondary screening formed decolorization circles on Congo red-cellulose.

Isolates		Decolorization zone diameter (mm)	Colony diameter (mm)	Ratio (D/d)
Bacteria	CB118	57.61±0.15d	2.10±0.21a	27.43±0.24c
	BD3	53.82±0.57c	6.34±0.50e	8.49±0.53a
	B58	52.15±0.11c	1.82±0.20a	28.65±0.17c
	B57	52.53±0.75c	2.03±0.21a	25.88±0.45c
	BN15	48.64±0.42b	1.63±0.12a	29.84±0.28c
	CB156	47.01±1.57b	5.02±0.42d	9.36±0.83ab
	YB3	48.95±0.14b	2.41±0.31ab	20.31±0.28bc
	CB179	49.3±0.26b	3.34±0.34b	14.76±0.30b
	CB161	50.17±0.19b	2.60±0.14ab	19.30±0.17b
	CB13	44.12±0.21a	3.04±0.22b	14.51±0.22b
Fungi	FB1	23.87±0.12c	6.42±0.10e	3.72±028a
	FB2	22.74±0.18c	6.21±0.44e	3.66±0.31a
	FZ8	23.76±0.21c	4.06±0.23b	5.85±0.22a
	FH2	23.54±0.01c	3.42±0.24b	6.88±0.14a
	FH3	20.81±0.22b	4.85±0.18c	4.29±0.19a
	FH5	23.02±0.57c	5.24±0.51d	4.39±0.53a
	FT1	8.40±0.12a	4.24±0.35c	1.98±0.27a
	FF1	22.22±0.55c	5.24±0.42d	4.24±0.48a
	FF2	20.04±0.07b	3.52±0.27b	5.69±0.16a
	FF3	20.81±0.21b	4.35±0.16c	4.78±0.20a

Note:D: The diameter of transparent zone; d: The diameter of colony; Ratio = D/d.

Supplementary Table S3. The strains selected for secondary screening formed decolorization circles on aniline blue media.

Isolates		Decolorization zone diameter (mm)	Colony diameter (mm)	Ratio (D/d)
Bacteria	CB118	19.84±0.12a	5.57±0.05b	3.56±0.03b
	BD3	38.91±0.86d	5.98±0.40b	6.51±0.59d
	B58	37.46±0.52d	5.01±0.43b	5.01±0.22c
	B57	28.93±0.42c	4.51±0.14a	4.51±0.16c
	BN15	58.74±0.27g	11.34±0.16c	5.18 ±0.05c
	CB156	23.38±0.14b	4.84±0.59a	4.83±0.59c
	YB3	52.39±0.22f	19.31±0.33d	2.71±0.04b
	CB179	66.4±0.95h	35.91±0.45e	1.85±0.03a
	CB161	45.55±0.25e	13.92±0.11c	3.27±0.04b
	CB13	45.62±0.49e	13.27±0.11c	3.44±0.01b
Fungi	FB1	40.07±0.17c	12.84±0.08b	3.12±0.02b
	FB2	68.84±0.06f	33.87±0.19d	2.03±0.04a
	FZ8	65.4±0.58f	23.59±0.69c	2.77±0.07a
	FH2	17.38±0.43a	5.39±0.64a	3.22±0.14b
	FH3	18.04±0.15a	4.85±0.91a	3.72±0.25c
	FH5	45.97±0.60d	11.5±0.95b	4.00±0.24cd
	FT1	16.35±0.29a	4.34±0.73a	3.77±0.45c
	FF1	32.07±1.32b	11.09±0.28b	2.89±0.48ab
	FF2	48.08±0.90d	12.24±0.22b	3.93±0.08c
	FF3	55.37±0.26e	22.56±0.81c	2.45±0.01a

Note: D: The diameter of transparent zone; d: The diameter of colony; Ratio = D/d.

Supplementary Table S4. Inhibition rates of partial strains against rice pathogens

The tested isolates	Inhibition rate（%）				
	CB13	CB118	CB156	BD3	BN15
Ustilaginoidea virens	96.49±0.32	94.52±0.33	88.76±0.24	93.83±0.34	85.37±0.41
Magnaporthe oryza	92.68±0.14	91.82±0.16	88.56±0.27	89.27±0.38	91.81±0.32
Rhizoctonia solani	66.33±0.23	43.86±0.36	23.58±0.21	32.74±0.25	56.17±0.38
Fusarium fujikuroi	69.62±0.23	64.26±0.17	38.53±0.15	41.82±0.41	63.65±0.28

Note: mean ± SD, n=3 biological replicates

Supplementary Table S5. Antagonistic test among candidate strains

	**CB13**	**CB118**	**CB156**	**BD3**	**BN15**	**PY1**	**FH2**	**FH5**	**FF2**	**FF3**
**CB13**		×	√	√	√	×	×	×	×	×
**CB118**	×		√	√	√	×	×	×	×	×
**CB156**	√	√		√	√	×	×	×	√	√
**BD3**	√	√	√		√	×	×	×	×	×
**BN15**	√	√	√	√		×	×	×	×	×
**PY1**	×	×	×	×	×		×	×	×	×
**FH2**	×	×	√	×	×	×		√	√	√
**FH5**	×	×	√	×	×	×	√		√	√
**FF2**	×	×	×	×	×	×	√	√		√
**FF3**	×	×	×	×	×	×	√	√	√	

Note: √: The strains can be co-cultured without antagonistic effects; ×: The strains cannot be co-cultured due to the presence of antagonistic interactions.
